# COVID-19 seroprevalence after the first UK wave of the pandemic and its association with the physical and mental wellbeing of secondary care healthcare workers

**DOI:** 10.1016/j.bbih.2022.100492

**Published:** 2022-08-06

**Authors:** Sonika Sethi, Natasha Manuelpillai, Anandadeep Mandal, Olivia Simpson, Hana Morrissey, Patrick Ball, Hayley Sharrod-Cole, Clare Ford, Anna C. Whittaker, Mark Drayson, Adam Race, James Bateman, Supratik Basu, James Cotton

**Affiliations:** a The Royal Wolverhampton NHS Trust, Wolverhampton, UK; b Sandwell and West Birmingham NHS Trust, Birmingham, UK; c Isle of Wight NHS Trust, Isle of Wight, UK; d University of Birmingham, Birmingham, UK; e University of Wolverhampton, UK; f National Institute for Health Research, Clinical Research Network, West Midlands, UK; g Research Institute in Healthcare Sciences (RIHS), University of Wolverhampton, Wolverhampton, UK; h Aston Medical School, UK; i Faculty of Health Sciences and Sport, University of Stirling, Stirling, UK

**Keywords:** SARS-CoV-2, COVID-19, GAD-7©, SF-12©, Frontline workers, Healthcare professionals, Anxiety, Resilience, Physical health, Mental health and wellbeing

## Abstract

**Objectives:**

To determine the seroprevalence of severe acute respiratory syndrome-coronavirus-2 (SARS-CoV-2) antibody status amongst healthcare workers (HCWs) working through the first wave of the Coronavirus (COVID-19) pandemic in 2020. To examine the association of seroprevalence and self-reported COVID-19 symptoms with occupation, sex, and ethnicity; and how these factors were associated with physical and mental wellbeing.

**Design:**

Single-centre cohort study.

**Setting:**

Large public hospital in the United Kingdom.

**Intervention:**

All HCWs who had been tested for anti-SARS-CoV-2 immunoglobulin (Ig) G nucleocapsid antibody in summer 2020 were asked to complete an electronic survey focusing on their physical and mental health in Winter 2020–21. This survey was comprised of the Short Form 12v2, Physical Component Summary (PCS), Mental Component Summary (MCS), and Generalised Anxiety Disorder 7-item (GAD-7) questionnaires.

**Results:**

7604/9781 (77.7%) HCWs were antibody tested, of which 1082 completed the full survey. Antibody testing was conducted between 17/06/20–30/07/20, during which time our seroprevalence rate was 28% (299/1082). Of those self-reporting COVID-19 symptoms, 51% (201/395) were antibody positive. Antibody-positive participants had lower PCS scores (p = 0.016), indicating poorer physical health. Lower PCS scores were also found in those deemed high risk for COVID-19 by their GP (p = 0.001), and those aged >44 years (p = 0.009). Antibody-negative participants had lower MCS scores (p = 0.044), indicating poorer mental health. Those who self-reported COVID-19 symptoms had lower PCS scores (p=<0.001) than those with no symptoms. Lower MCS scores were found in women (p = 0.001), Caucasians (p = 0.018), non-clinicians (p = 0.001), and those aged <44 years (p = 0.009). Significantly higher GAD-7 anxiety scores were evident in staff aged <44 years (p = 0.023), and those with self-reported COVID symptoms (p = 0.031). Doctors had lower GAD-7 anxiety scores (p = 0.009).

**Conclusion:**

Self-reported symptoms did not correlate with seroprevalence; data surrounding this can be useful for future workforce planning. Interventions are needed to reduce the mental and physical burden of the pandemic on HCWs. Further work is needed to identify which particular HCWs may require further support, to ensure well-being and effective patient care.

**Trial registration:**

Sponsor Protocol number - 2020COV112, Clinicaltrials.gov number -NCT04527432.

## Highlights of this study

• Large study examining COVID-19 seroprevalence in healthcare workers (HCWs).
• Links seroprevalence to physical and mental health-related quality of life scores.
• Responses capture the impact from the first COVID-19 wave in the United Kingdom.
• Set in an ethnically diverse National Health Service (NHS) Trust including all staff groups.

## 1 Introduction

The Coronavirus disease 2019 (COVID-19) pandemic has presented unprecedented challenges to healthcare systems globally as a result of its rapid spread and limited effective treatments for it (Blumenthal et al., 2020). The COVID-19 pandemic has amplified the risks and challenges faced by healthcare workers (HCWs), with increased pressure, workload and personal uncertainty (Vera San Juan et al., 2021). However, the impact on the physical and mental health of staff across secondary care centres remains poorly understood (Mehta et al., 2021) but this information could be vital for future pandemic response planning (Holmes et al., 2020). The understanding of infection rates in HCWs is evolving, with a large linked UK cohort study suggesting a two-to three-fold risk of hospital admission with COVID-19 for HCWs, although absolute risk remains relatively low (Shah et al., 2020). Data related to infection rates in HCWs have largely relied on the real-time reverse transcriptase-polymerase chain reaction (RT-PCR) test, rather than determining seroprevalence using severe acute respiratory syndrome coronavirus 2 (SARS-CoV-2)nucleocapsid antibodies. These antibodies indicate previous infection when measured at least two weeks after the onset of symptoms (Halili et al., 2022).

Several research studies and meta-analyses highlight the risks of depression, anxiety and insomnia in HCWs during the COVID-19 pandemic, but less is known about longer-term physical and mental health implications (Pappa et al., 2020; Shortfall of 50,000 doctors may overwhelm NHS in winter, 2021). There are concerns that these effects will lead to even more staff leaving the hospital workforce, eventually leading to a shortfall in all grades of staff (Shortfall of 50,000 doctors may overwhelm NHS in winter, 2021). COVID-19 has been shown to disproportionately affect Black, Indigenous, and people of colour (BIPOC) patients, although research in this cohort for both patients and HCWs is previously lacking (Pan et al., 2020; Treweek et al., 2020; Eyre et al., 2020). Research in learning about the antibody response and its impact on re-infection rates is on-going in both single centre and multi-centre trials. However, there is a lack of knowledge of how the physical or mental health of HCWs has been affected by the COVID-19 pandemic and how this could vary by demographic factors, infection risk and, in particular, antibody status (Lumley et al., 2021; Hall et al., 2021).

The COVID-19 Health Professional Impact Study (CHIP) study is a single centre cohort study, evaluating the impact of COVID-19 on the physical and mental health of a National Health Service (NHS) hospital workforce and the association between these and the presence of SARS-CoV-2 nucleocapsid antibodies, and the demographics and job types related to this.

### 1.1 Aims and objectives

There were two main aims of this study. Firstly, to determine the prevalence of SARS-CoV-2 antibodies in workers between 17th of June to July 30, 2020 (first wave of the pandemic), and how this compares to symptomatic self-reported COVID-19 infection. Secondly, to identify whether physical and mental health, including anxiety levels of HCWs relate to SARS-CoV-2 nucleocapsid antibody status, sex, age, ethnicity, job role (patient-facing or not, or if a doctor), high risk COVID-19 status (as classified by their GP, specialist or occupational health due to their health problems), and having symptoms of COVID-19.

## 2 Methods

### 2.1 Study participants

This study was conducted at The Royal Wolverhampton NHS Trust (RWT), West Midlands, UK. In March 2020, RWT employed 9871 staff covering over 350 roles across three hospitals, including an 850 bedded secondary and tertiary care centre (Equalities Information, 2021; About Us, 2021). Wolverhampton is one of the most ethnically diverse areas in the UK, which is closely reflected in RWT's work force. 31% of the RWT workforce are BIPOC and the majority of the BIPOC staff (62%), are in the medical and dental departments (Equalities Information, 2021). Further, Wolverhampton's population is within the highest decile of deprivation in the UK and was one of the earliest and hardest hit areas from COVID-19, reaching a total of 24,516 cases by March 2021 (local population approximately 260,000). (Public-Health-Annual-Report-2020-21., 2020).

### 2.2 Study design

**Fig. 1:**
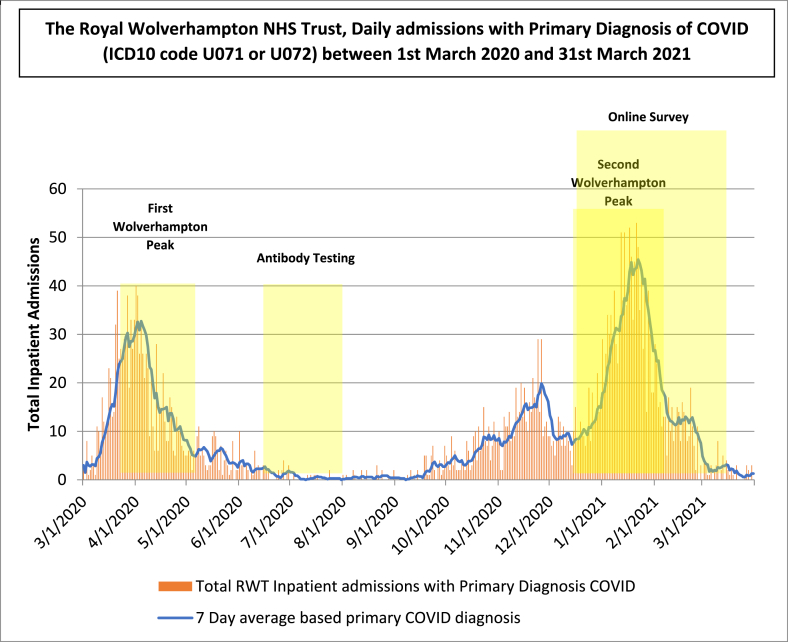
Infographic timeline of the study in relation to the COVID-19 pandemic in Wolverhampton Note: Can be kept black and white.

All HCWs which included both clinical and non-clinical staff (support staff and estates teams included) were invited to participate in SARS-CoV-2 nucleocapsid antibody testing between 17th of June to July 30, 2020 (Fig. 1). Results are reported by dividing the sample response by the stored calibrator response. The default result unit for the SARS-CoV-2 IgG anti-nucleocapsid antibody assay is Index (S/C). A result ≥1.4 Index (S/C) is considered to be positive. Antibody testing was performed using the Abbott SARS-CoV-2 assay that detects Immunoglobulin (Ig) G anti-nucleocapsid antibodies; analysed on either Abbott Architect i2000sr or Alinity ci (Abbott Diagnostics, Abbott Park, IL, USA). The Abbott assay was evaluated by the Clinical Service unit at PHE Colindale between the 4th and May 7, 2020. The assay specificity was 99.73% which is in accordance with the manufacturer's reported specificity of 99.63%. The assay gave an overall sensitivity of 92.71% with a sensitivity ≥14 days from infection of 93.90%. In-house verification of the assay demonstrated a sensitivity of 91.1% (95% CI 83.2–96.1) and specificity of 100% (95% CI 94.1–100) (Evaluation of the Abbott SARS, 2022). As part of antibody testing, staff provided a mobile phone contact number and electronic consent using a smartphone or computer. SARS-CoV-2 nucleocapsid antibody status (positive or negative status) was sent back to staff via a Short Message Service (SMS).

This health survey component of the study ran from December 16, 2020 to March 15, 2021 (Fig. 1). We invited participation via three methods, each of which linked to an online participant invitation and information sheet and online electronic consent form (Appendix A). Firstly, smartphone-based invitations, using a novel mobile SMS invitation, as previously described, were distributed on December 16, 2020 with a reminder January 2, 2021 (Cleaton et al., 2021). Secondly, we invited participants via an email-based staff bulletin. Thirdly, we used a Quick Response (QR) code displayed on posters and computer screensavers around our hospital, including staff vaccination areas, which directly accessed the study website. Inviting all staff members via individualised SMS and virtual reach enabled those unable to attend for individual face to face consent to participate, and thus more opportunity to be represented, in comparison to larger multi-centre face to face research studies (Lumley et al., 2021). Data for participants in this analysis were included from December 16, 2020 to March 15, 2021. Fig. 1 is an infographic showing a timeline of our study alongside the COVID-19 pandemic and COVID-19 admissions in RWT.

For data management and linkage, unique identifiers in the form of a mobile phone number or employee number were used to allow us to link SARS-CoV-2 nucleocapsid antibody status anonymously. Staff completed a web-based survey (appendix A). This included information on demographics, including age, sex, ethnicity, high risk COVID-19 status (as determined by the Trust occupational health team, GP or specialist assessment), occupation and whether this role was patient-facing or not, occurrence and dates of experiencing symptomatic COVID-19 infection as defined by the World Health Organisation (The British Medical Association, 2021), as COVID-19 infection accuracy was limited by a lack of access to polymerase chain reaction (PCR) testing in the early part of the pandemic. It is worth noting that hospital workers were required to self-isolate for 14-days as a minimum following onset of COVID, and could only test on return, allowing a minimum 2-week spell post infection to antibody status. Some participants contracted COVID-19 following antibody testing; this group is described separately in the tables.

Participants completed the Short Form 12v2 (SF12v2) (Yin et al., 2016) and the Generalised Anxiety Disorder 7-item (GAD-7) (Spitzer et al., 2006). The SF12v2 allows the calculation of physical health (Physical Component Summary, PCS) (PCS) and mental health (Mental Component Summary, MCS), each on a 0–100 scale (0 being the lowest quality of physical and mental health) (Yin et al., 2016). These scores are internationally recognised as high quality measurements of mental and physical wellbeing, known as health related quality of life (HRQoL) (Ware et al., 1996). A minimally important clinical difference (MICD) of 2 points for SF12v2 was used for our study (Maruish, 2012). The GAD-7 is a self-assessment tool reporting on a 0–21 scale, where a higher score indicates an increasing level of anxiety. It is a reliable score for diagnosing Generalised Anxiety Disorder (GAD) and indicating symptoms of anxiety (Spitzer et al., 2006). An MICD of 4 was used for GAD-7 (Löwe et al., 2008).

Data were collected in an anonymised manner and stored securely using SurveyMonkey (Survey Monkey Enterprise), a secure survey tool compliant with the Health Insurance Portability and Accountability Act (HIPAA). (HIPAA Compliant Survey Software, 2021).

### 2.3 Statistical analysis

The statistical analysis was performed in four phases. The first phase described the data of the participants in this COVID-19 survey, including the various factors considered in the analysis. Observations for SARS-CoV-2 seroprevalence, symptomatic COVID-19, and HRQoL were classified based on the following fixed covariates: sex, age, ethnicity, job role, patient-facing role, and high-risk status, observed as categorical data. Descriptive statistics for the quantitative variables are presented as mean for PCS, MCS, and GAD-7. Descriptive statistics for categorical variables are presented as frequency. In the second phase we conduct independent samples *t*-test to examine whether there were significant differences in the PCS, MCS and GAD-7 scores between those with versus without self-reported COVID-19 infection and COVID-19 antibody-positive participants versus negative participants. The third phase explored any significant differences in the PCS, MCS and GAD scores by each of the above-mentioned demographic classifications using independent samples *t*-test; from which we also report the effect size as Cohen's D measure. The fourth phase examined whether PCS, MCS, and GAD-7 scores were significantly influenced by key demographics and classifications in a stepwise multiple regression model is used to examine this. We used dummy coding for the categorical independent variables that included Gender (Female), Ethnicity (Caucasian), Role (Doctor), COVID-19 Symptom (Self-Reported), Antibody Test (Positive), Patient Facing Role. All analyses were carried out in STATA® software version 16.1 (StataCorp, 2019).

### Ethical approval

Ethical approval was granted by the East of England – Cambridge South Research Ethics Committee under reference 20/EE/0201 and Integrated Research Application System (IRAS) number 287432 (appendix B). The study protocol (appendix C) is registered with Clinicaltrials.gov number – NCT04527432.

### Role of the funding source

The trial sponsors were RWT and the 10.13039/501100000272National Institute for Health Research (NIHR) Clinical Research Network West Midlands. The Abbott SARS-CoV-2 IgG anti-nucleocapsid antibody test kits were provided free of charge. The sponsor, funder and Abbot laboratories had no role in study design, analysis or reporting of this study.

## 3 Results

**Fig. 2:**
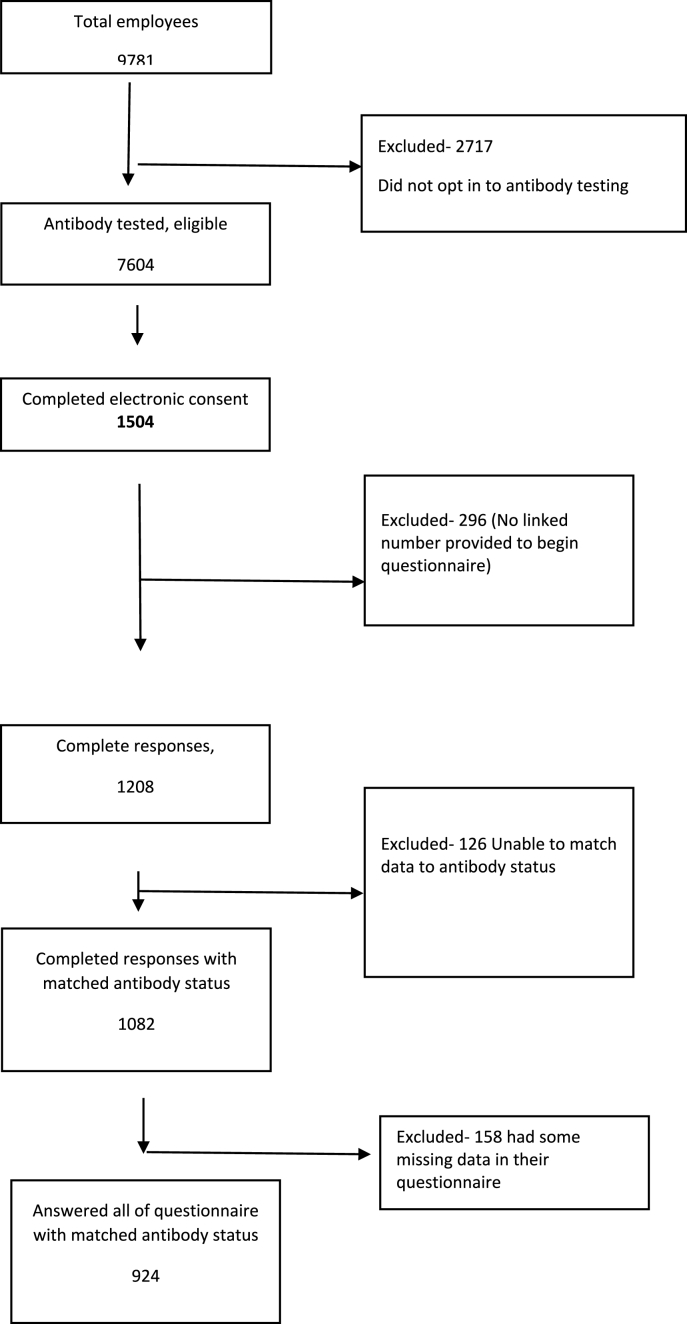
Study profile.

In March 2020, there were 9781 employees at RWT of which 7604 (77.7%) were tested for antibodies and were eligible for inclusion (Fig. 2). 1504 HCWs (19.8% of tested staff) were consented to participate and asked to provide a mobile phone/employee number to allow linkage of data, of whom 1208 (80.3%) did and completed the study survey. However, a further 126 were excluded as we were unable to match data with their antibody status. 1082 participants had matched survey data with antibody status and of this group, 924 had fully completed the survey. (Fig. 2). The participant information sheet had informed participants that they could omit answering certain questions if they were not emotionally prepared to do so.

Baseline characteristics of all respondents are shown in Table 1. The majority of respondents were female (84.4%), of white ethnicity (85.2%), stated that they had a patient-facing role (53.2%). The age band decade with the highest number of participants was 45–54 years old (27.9%) and nearly half all respondents were nurses (46.9%). The BIPOC community (14.8%) included those of Asian (9.7%), Black (2.2%), Mixed (1.0%), Chinese (0.6%) and ‘Other’ ethnicities (1.2%). Other roles included administrative/manager/clinical scientist (28.9%), volunteers (10.6%), doctors (9.7%) and other support staff (3.8%). 5.8% were considered high risk patients from COVID-19 infection, classified by their GP, specialist, or the hospital occupational health service (Table 1).

**Table 1:** Data summary.

**All participants responding to questionnaire**		Totals	Seroprevalence SARS-CoV-2			Symptomatic COVID*		HRQOL		
			Positive	Negative	Missing	Yes	No	PCS	MCS	GAD7
		1208	299	783	126	441	767	55.56	44.13	5.43
**Gender**										
	**Male**	164	36	104	24	45	119	55.98	44.12	5.45
	**Female**	1044	248	694	102	396	648	55.50	44.14	5.43
**Age**										
	**Under 18**	4	1	2	1	3	1	58.40	48.83	2.50
	**18–24**	41	11	24	6	16	25	59.35	40.12	7.39
	**25–34**	220	54	136	30	95	125	57.47	42.58	5.81
	**35–44**	278	62	183	33	105	173	56.71	42.89	5.63
	**45–54**	337	79	232	26	123	214	54.67	44.33	5.47
	**55–64**	282	66	189	27	89	193	54.06	46.35	4.62
	**Above 65**	46	11	32	3	10	36	51.59	47.24	5.67
**Ethnicity**										
	**Asian**	118	33	68	17	51	67	54.90	46.05	4.95
	**Black**	27	2	19	6	10	17	54.58	47.27	4.85
	**White**	1029	236	693	100	365	664	55.71	43.75	5.52
	**Mixed ethnicity**	12	6	5	1	4	8	53.92	45.78	5.50
	**Chinese**	7	3	3	1	4	3	53.31	51.32	3.71
	**Others**	15	5	10	0	7	8	54.79	44.95	4.93
**Role**										
	**Nursing, HCA, AHP**	566	157	356	53	228	338	55.44	43.88	5.62
	**Doctor**	118	33	65	20	57	61	56.90	47.50	3.97
	**Other support (porter, cleaner, security, domestic, estates)**	46	20	20	6	15	31	54.12	46.42	4.83
	**Administrative, manager, clinical scientist**	349	50	269	30	100	249	55.91	43.00	5.72
	**Volunteer**	129	24	88	17	41	88	54.44	44.42	5.39
**Patient Facing Role**										
	**Yes**	643	191	389	63	277	366	55.81	44.29	5.44
	**No**	565	93	409	63	164	401	55.28	43.95	5.42
**High risk Group for COVID**										
	**Yes**	70	25	37	8	70	0	49.83	40.98	6.73
	**No**	371	176	157	38	371	0	54.73	43.83	5.38
	**Missing**	767								

1082 (89.7%) survey respondents were linked to their SARS-CoV-2 nucleocapsid antibody status. 395 (36.5%) of these had self-reported COVID-19 symptoms, of which 51% were, in fact antibody positive. The overall SARS-CoV-2 nucleocapsid antibody seroprevalence rate was 27.6% as of July 30, 2020. Out of participants who were seropositive, 67% had self-reported COVID-19 infection at or before the time of the survey (Table 2).

**Table 2:** Seroprevalence and HRQOL; PCS; physical component score. MCS; Mental Component Score. GAD 7; general anxiety disorder assessment.

			Seroprevalence SARS-CoV-2		HRQOL		
			Positive	Negative	PCS	MCS	GAD7
**Participants with linked Antibody Data**							
	Totals	1082	299	783	55.67	43.70	5.81
**Self-reported COVID-19 Infection**							
	No COVID-19 infection	687	83	604	56.37	44.52	5.40
	COVID-19 infection at any time	395	201	194	53.84	43.07	5.79
	Significance (p-value)				(0.041)*	(0.507)	(0.419)
	Cohen's D				0.381	0.126	−0.046
**COVID-19 Positive patients only**							
	Symptomatic COVID-19 **before** SARS-CoV-2 antibody testing	237			54.16	43.44	5.51
	Symptomatic COVID-19 **post** SARS-CoV-2 antibody testing	158			53.36	42.53	6.22
	Significance (p-value)				(0.510)	(0.458)	(0.031)*
	Cohen's D				0.178	0.115	−0.311

Note: *indicates significance at 5% level The values in parenthesis are the *p*-values of the independent sample *t*-test. The effect size is measured by Cohen's D estimate.

Of the HRQoL scores in Table 2, PCS was significantly higher in those who had no symptomatic COVID-19 infection compared to those reporting COVID-19 symptoms at the time of the survey (mean difference +/−, p = 0.041) showing a medium effect size, estimated using Cohen's D measure. There was a significant difference in anxiety (higher GAD-7 score) in those who reported COVID-19 symptoms after antibody testing compared to those having symptoms before testing (mean difference +/−, *p* = 0.031). The Cohen's D estimate showed medium effect for the significant difference in the GAD-7 scores.

924 (85%) participants completed the survey fully and could be linked to antibody data (Table 3). Regression was used for the factors that could influence PCS, MCS and GAD-7 (Table 4). PCS was significantly higher in those who: were negative for SARS-CoV-2 nucleocapsid antibodies (*p* = 0.016); those with no symptoms of COVID-19 infection compared to those with symptoms (*p* = 0.009); those 44 years or younger (*p* = 0.009); and those not in the high-risk group for COVID-19 (*p* = 0.001) (Table 3, Table 4). PCS scores were significantly lower in those with past or present COVID-19 infection compared to those without.

**Table 3:** Mental and Physical Health related to various factors.

		Observations	PCS	MCS	GAD7
All participants with complete linked data	Total	924	55.80	44.24	5.43
Antibody status					
	Positive	245	54.83	45.29	4.97
	Negative	679	56.15	43.85	5.60
	Significance (p-value)		(0.016)*	(0.044)*	(0.068)
	Cohen's D		−0.380	0.311	−0.116
Self-reported COVID Symptoms					
	Symptomatic COVID-19 infection	239	54.16	43.44	5.51
	No symptoms of COVID-19 infection	685	56.36	44.52	5.40
	Significance (p-value)		(0.009)*	(0.138)	(0.137)
	Cohen's D		−0.318	−0.116	0.018
Gender					
	Male	125	56.40	46.94	4.05
	Female	799	55.71	43.83	5.63
	Significance (p-value)		(0.323)	(0.001)*	(0.001)*
	Cohen's D		0.017	0.413	−0.331
Age					
	44 or younger	399	57.63	42.35	5.84
	45 or above	525	54.38	45.71	5.11
	Significance (p-value)		(0.009)*	(0.009)*	(0.023)*
	Cohen's D		0.501	−0.331	0.301
Ethnicity					
	Caucasian	803	55.96	43.95	5.48
	non-Caucasian	121	54.56	46.19	5.09
	Significance (p-value)		(0.054)	(0.018)*	(0.412)
	Cohen's D		0.308	−0.411	0.118
Role					
	Doctors	78	56.87	48.03	3.74
	non-Doctors	846	55.68	43.88	5.58
	Significance (p-value)		(0.166)	(0.001)*	(0.009)*
	Cohen's D		0.018	0.613	−0.481
Patient Facing Role					
	Yes	485	55.98	43.94	5.61
	No	439	55.61	44.56	5.23
	Significance (p-value)		(0.518)	(0.338)	(0.237)
	Cohen's D		0.009	−0.118	0.011
High-risk Group for COVID					
	Yes	38	50.23	43.23	5.50
	No	201	54.91	43.47	5.52
	Significance (p-value)		(0.001)*	(0.889)	(0.987)
	Cohen's D		−0.481	−0.011	0.011
	Not recorded	685			

Note: *indicates significance at 5% level The values in parenthesis are the *p*-values of the independent sample *t*-test. The effect size is measured by Cohen's D estimate.

**Table 4:** Factors affecting Physical Component Summary (PCS), Mental Component Summary (MCS) and Generalised Anxiety Disorder assessment GAD-7 scores.

Factors	PCS		MCS		GAD	
	Coefficient	p-value	Coefficient	p-value	Coefficient	p-value
Gender (Female)	−0.712	(0.323)	**−3.106**	(0.001)*	**1.587**	(0.001)*
Ethnicity (Caucasian)	**1.397**	(0.054)	**−2.238**	(0.018)*	0.389	(0.412)
Role (Doctor)	1.019	(0.166)	**4.151**	(0.000)*	**−1.843**	(0.001)*
Covid Symptom (Self-Reported)	**−2.231**	(0.000)*	−1.080	(0.138)	0.085	(0.815)
Antibody Test (Positive)	**−1.318**	(0.016)*	**1.446**	(0.044)*	**−0.655**	(0.068)
Patient Facing Role	0.314	(0.518)	−0.622	(0.338)	0.377	(0.237)

Note: *indicates significance at 5% level. The regression is run on 924 participants. The table shows the impact of various categorical variables on PCS, MCS and GAD-7. The various factors included are: Gender (Female), Ethnicity (Caucasian), Role (Doctor), Covid Symptom (Self-Reported), Antibody Test (Positive), Patient Facing Role. A negative correlation indicates a better outcome in the score. Higher scores indicate better PCS and MCS but worse GAD7.

MCS scores were significantly lower in those who had negative antibody status (*p* = 0.044), female staff (*p* = 0.001), people 44 years and below (*p* = 0.009), Caucasians (*p* = 0.018) and non-Doctors (*p* = 0.001) (Table 3). GAD-7 was significantly higher in females (*p* = 0.001), people 44 years or below (*p* = 0.023) and non-doctors (*p* = 0.009) (Table 3, Table 4). Despite PCS being lower for those high risk for COVID-19, there was no significant difference in MCS and GAD-7. The Cohen's D estimate shows that all the effect size of the significant values are medium.

## 4 Discussion

Whilst the risks of COVID-19 infection in HCWs have been widely described, the CHIP study is one of the first, and largest, in linking symptomatic infection, antibody positivity and mental and physical HRQoL scores (Petzold et al., 2020).

### 4.1 Physical and mental health

Individuals who were antibody positive or symptomatic had poorer self-reported physical health; this is consistent with data showing that those with poorer physical health are more at risk of contracting COVID-19 (Evans et al., 2021, Shanbehzadeh et al., 2021) but may also result from the impact of COVID-19 infection on self-reported health (Sanyaolu et al., 2020). Whilst a study has already found that PCS scores were worse in patients post COVID-19 (Evans et al., 2021, Shanbehzadeh et al., 2021), this has now also been found in a HCW population (Sanyaolu et al., 2020; O’Kelly et al., 2022). Poorer physical health was found in high risk HCWs, which can be related to their co-morbidities predisposing them to a greater risk of symptomatic infection with COVID-19 (Sanyaolu et al., 2020), although direction of causality for this cannot be claimed from observational data even in longitudinal studies. Physical health was better in those that were younger and without health conditions, as found in other studies (Sanyaolu et al., 2020).

Compared to current normative data, the mental health of the HCWs in our cohort was worse (Ware et al., 1996). Our study supports several other studies that show that mental health of HCWs has been negatively impacted from the COVID-19 pandemic (de Kock et al., 2021; Fernandez et al., 2021; Tiete et al., 2021). Reasons may include an increased workload, a greater risk of contracting COVID-19 than the general public, fear of transmitting COVID-19 to friends and family, physical exhaustion, social isolation, negative impact of lockdown, understaffing due to sickness, longer working hours and anxieties related to the wearing of personal protective equipment (PPE) (Vera San Juan et al., 2021; de Kock et al., 2021; Fernandez et al., 2021; Tiete et al., 2021; Naqvi et al., 2021; Temsah et al., 2020; Karlsson and Fraenkel, 2020).). Whilst poorer mental health was seen throughout the pandemic, it is difficult to link causally to COVID-19 infection, owing to the many, varied other factors associated with the pandemic that could have an impact on an individual's mental well-being. Hamilton et al. (2021), however, found that higher levels of inflammation seen in COVID-19 increase the vulnerability of older people to impaired mental health. An observational study by Magnusdottir et al. (Magnúsdóttir et al., 2022) also found that severe acute COVID-19 illness is associated with long term mental health morbidity.

In our study, those who were seropositive had significantly better mental health compared to those who were seronegative, which supports the findings of other investigations that have demonstrated a reduction in anxiety levels once an individual has contracted and subsequently recovered from the virus (Vera San Juan et al., 2021). Intriguingly, some studies have shown that higher stress and anxiety levels are associated with delayed, weaker peak antibody levels following SARS Cov-2 infection, and also shorter-lived immune responses to vaccination (Glaser and Kiecolt-Glaser, 2005; Pedersen et al., 2009; Madison et al., 2021; Phillips, 2011).

This current study also reinforces that females experienced worse mental health during the COVID-19 pandemic and similar results have been found in other studies (Çelmeçe and Menekay, 2020; Shen et al., 2020; Hubbard et al., 2021; Padovan-Neto et al., 2021; Jafri et al., 2022), with women having a higher prevalence of mood disorders, specifically depression and anxiety ((de Kock et al., 2021; Xiong et al., 2020; Luceño-Moreno et al., 2020)). However, a greater majority of our study population consisted of women. Younger people tend to generally have better physical health, yet ours and several studies have suggested COVID-19 anxiety was higher among younger adults than their counterparts (de Kock et al., 2021; Tiete et al., 2021; Hubbard et al., 2021; Jia et al., 2020; Nwachukwu et al., 2020). Doctors were the least anxious compared to the non-medical and nursing staff, which has been supported in other studies (de Kock et al., 2021; Fernandez et al., 2021; Tiete et al., 2021; Çelmeçe and Menekay, 2020; Shen et al., 2020; Vizheh et al., 2020; Siddiqui et al., 2021; Zhang et al., 2021).

### 4.2 Seroprevalence & symptomatic COVID-19

Having an awareness of seroprevalence can be useful from a workforce planning perspective and in reviewing infection control measures. Seroprevalence in other studies in HCWs greatly vary; the majority being between 11% and 33% and the highest rates being in New York and London (Eyre et al., 2020; Grant et al., 2021; Mansour et al., 2020). In comparison with another study conducted in the West Midlands, our seroprevalence rate (28%) was higher by 3%, with a sample size nearly double of the other study (Shields et al., 2020). The seroprevalence rate in the West Midlands general population at the time of sampling was 7% (National COVID, 2021). Our study, in common with others, found that seroprevalence is much greater in HCWs than in the general population (Eyre et al., 2020; Grant et al., 2021; Mansour et al., 2020).

Despite the higher seroprevalence in HCWs than the general population, there is a need to confer long lasting immunity through vaccinations rather than relying on natural infection (Shields et al., 2020). Seroprevalence can vary greatly with time; Bendavid et al. (2021) showed that seroprevalence rate in participants two weeks after symptoms was considerably higher than its rate three months after symptoms (Pedersen et al., 2009; Bendavid et al., 2021). The duration for which antibody levels remain elevated is currently unknown (Hall et al., 2021). The low amount of positive antibody tests in comparison with symptomatic infection could be due to antibody loss at the time of testing or not mounting enough of an immunological response when symptomatic (Halili et al., 2022). As stated above, higher stress and anxiety levels can influence the timing, size and longevity of antibody responses (Glaser and Kiecolt-Glaser, 2005; Pedersen et al., 2009; Madison et al., 2021; Phillips, 2011). However, it is worth noting that health is a known type of anxiety on the spectrum of obsessive-compulsive disorder (OCD), this may have been present in some participants even prior to COVID-19 and perhaps even worsened. This aspect was impossible to account for considering that anxiety is the most prevalent mood disorder globally and in the UK- yet is still underrecognized and thus undertreated (Bendavid et al., 2021; Bandelow and Michaelis, 2015; Kasper, 2006; The National Institute for Health and Care Excellence, 2011; World Health Organization, 2019).

### 4.3 The future

As the on-going pressures of COVID-19 within our communities become evident, understanding the mental and physical health impact of the pandemic will become increasingly important. The UK already has a lower doctor to population ratio and lower hospital bed per capita rate than many other European nations (Shortfall of 50,000 doctors may overwhelm NHS in winter, 2021; The British Medical Association, 2021; Rocks and Boccarini, 2021; Robertson, 2017; Moberly, 2017; Papanicolas et al., 2019). Furthermore, the UK and has a lower gross domestic product (GDP) percentage spend on healthcare compared to many high income EU nations. Staff illness (both physical and mental) may pose a greater risk to the delivery of healthcare in the UK, than in other comparable countries (Shortfall of 50,000 doctors may overwhelm NHS in winter, 2021). Addressing occupational and environmental factors will be key, along with teamwork and encouraging a stable social support network, to create a more resilient NHS (de Kock et al., 2021). Building knowledge on resilience and coping mechanisms will help in addition to the provision of adequate, appropriate PPE. HCWs should be able to seek psychological help without stigmatisation and will need to be carefully supported to ensure their ability to have a successful long-term career in the field (Petzold et al., 2020).

In terms of future work following vaccination, this study is being extended to capture further longitudinal data and to monitor the association between antibody positivity, COVID-19 infection rate and physical and mental HRQoL. During this study period, COVID-19 vaccination had only just begun, and antibodies were only tested prior to vaccination. In our extended study we will aim to address the impact of vaccination on seropositivity, mental and physical health scores and also the impact of physical and mental health on the antibody response to vaccination (Allen et al., 2022).

### 4.4 Limitations

We aimed to recruit all staff as widely as possible by providing them with an individual personal SMS message. This was to capture a key but under-represented population in research, which is relatively deprived and ethnically diverse (Equalities Information, 2021). However, there was self-selection sampling bias as participation in the survey was voluntary. The BIPOC population remained under-represented in this study, similar to other studies during the COVID-19 pandemic (Sethi et al., 2021). The demographic breakdown, with a large proportion of the study being white, female nurses, was largely representative of the Trust population and of other NHS trusts. This was similarly seen in another study in a similar setting in the Netherlands (Cleaton et al., 2021).

There was an inability to link participants’ wider electronic healthcare records to gain further demographic information. Some staff did not have their antibody results linked to their employee identification or mobile phone number. Staff without access to mobile smartphones would also have limited access, however, all staff are provided with IT and internet access on site. Further, the COVID-19 self-reported cohort is likely to include a number of cases related to other medical problems which were self-labelled as COVID-19. Although this is a limitation, the psychological and physical health impact of work loss, fear of case transmission, isolation and other factors would apply irrespective of case confirmation.

Furthermore, we did not have baseline assessment of the HRQoL measures collected when the antibodies were assessed, making it difficult to fully ascertain if the increase or decrease of scores were related to COVID-19 infection. There are no large scale HRQoL scores available specifically for HCWs and the SF12v2 is norm referenced against the British population. In terms of mental health impact from COVID-19, there were many other factors that could be impacting this. This includes the negative effects of lockdown, social isolation and a personal history of mental health problems.

We acknowledge that being vaccinated or not may constitute in assurance and relief from infection anxiety, but not depression. This was considered at the time of the writing of this manuscript, however, at the first phase of the study vaccination was not available. The emergence of new variants requires further research to plan new approaches.

## 5 Conclusion

The COVID-19 health professional impact study (CHIP) study examines in detail the impact the COVID-19 pandemic had on various subgroups of front-line healthcare workers (HCWs) in terms of mental and physical health. Future work will focus on subsequent COVID-19 waves and the vaccination stage.

Our results are consistent with other studies revealing a significant impact of COVID-19 on the mental health of HCWs and association with inflammatory changes related to mental health conditions. Our findings further add to this by providing additional unique information on physical health and seroprevalence.

Further research on how to combat the impact of COVID-19 is required, and we add to calls from other researchers in this area. Interventions are needed to address the long-term effects COVID-19 has had on the HCWs, who have and will continue to play a crucial role in fighting the pandemic.

## Author contributions

SS, NM and OS were involved in writing the study and manuscript revisions.

AM did the statistical analysis of the study and manuscript revisions.

HM, PB, AW and MD were involved in manuscript revisions.

HSC, CF, AR were involved in the running of the study and in manuscript revisions.

JB involved in protocol development, the running of the study, writing the study and in manuscript revisions.

SP and JC involved in the concept of the study, protocol development, running of the study and in manuscript revisions.

## Funding

This work was supported by National Institute of Health and Care Research (sponsor number 2020COV112). Abbott Laboratories provided the SARS-CoV-2 Immunoglobin (Ig) test kits used in this study.

## Declaration of competing interest

Abbott Laboratories UK provided the SARS-CoV-2 Ig test kits but had no role in the design or manuscript development of the study.

None of the authors have relevant conflicts of interest to declare.
