# Supplementary material for: COVID-19 seroprevalence after the first UK wave of the pandemic and its association with the physical and mental wellbeing of secondary care healthcare workers
Source: Brain Behav Immun Health. 2022 Aug 6;24:100492. doi: 10.1016/j.bbih.2022.100492 (PMC9355737; doi:10.1016/j.bbih.2022.100492)
Supplement: Multimedia component 1 [file mmc1.pdf]

The COVID 19 Health Professional Impact Study

Research studying the current level of SARS-CoV-2 antibody positivity and likely risk to healthcare workers in the UK to COVID-19 infection.

Have you received an Antibody test at RWT?

You can help us to:

- **Find out whether certain characteristics have an impact on the likelihood of infection**
- **Find out whether certain characteristics have an impact on antibody response and**
- **See if the presence of antibodies has an effect on potential future infection over a 12 month period**

Please take part in our study now.

About our study

The COVID-19 pandemic has had a huge impact on healthcare resources and staff in the UK. Understanding the key risk factors associated with infection amongst healthcare workers is essential for future pandemic response plans. Currently there are scarce data relating to the infection rates and associated factors amongst healthcare workers in the UK.

We will use the findings of this research to detail the current level of SARS-CoV-2 antibody positivity and thereby infer the likely risk to healthcare workers in the UK to COVID-19 infection. We will determine whether certain characteristics will have an impact on likelihood of infection and antibody response. Furthermore we will determine the impact of the presence of antibodies on the likelihood of future clinical infection over a 12 month period.

You can help by taking part in our research study.

This 15 minute online survey will ask NHS staff about their:

- **work environments;**
- **demographics and behavioural factors;**
- **mental health and wellbeing;**
- **ethnicity;**
- **symptoms.**

This survey will be linked to your current antibody test results and then we will contact you at 6 and 12 months' time to repeat the survey and the antibody test.

Taking part

You can participate if you are an RWT member of staff, who has had an antibody test here at the Trust already as part of standard protocol.

Information for participants

Participant information sheet. For further information before completing the survey, please download the information sheet.

Psychological support

If you feel you need some support for your mental health or wellbeing, please download a list of resources.

You can use this even if you decide not to take part in the study. We also recommend that you seek support via the Trust's occupational health department.

Take part in the study

Please complete our online consent form and initial survey now.

It takes approximately 15 minutes.

CONSENT FORM

* 1. I confirm that I have read and understood the information sheet Version 5 dated 15/09/2020 for the CHIP study.

☐ Select to confirm

* 2. I have had the opportunity to ask questions and all my questions have been answered to my satisfaction.

☐ Select to confirm

* 3. I understand that my participation is voluntary and that I am free to withdraw at any time, without giving any reason, without my medical care or legal rights being affected.

☐ Select to confirm

* 4. I agree for my data held with HR to be linked with my survey answers and COVID19 antibody test result for the study and I agree to provide my mobile number and ESR number in order for this to be conducted.

☐ Yes

* 5. I give permission for Abbott and Abbott's representatives to receive copies of de-identified Study results.

☐ Select to confirm

* 6. OPTIONAL: I agree for my data collected for this study to be used for future research.

◯ Yes

◯ No

* 7. OPTIONAL A): I agree to have 2 optional extra blood tests for antibody testing at 6 and 12 months' time. [If you do not want to be part of this, you can still participate in the study - please continue with the survey - you will not be contacted for further tests]

◯ Yes

◯ No

* 8. OPTIONAL B) I agree to provide my NHS Number in order to link the optional antibody test results to the study data.

◯ Yes

◯ No

* 9. I agree to take part in the above study.

- Yes
- No

* 10. Please provide your mobile phone number below to allow us to link your responses to your COVID19 test result (essential)

Mobile Phone Number ______

11. Please tell us your ESR number (if unknown, leave blank)

______

* 12. Gender:

- ◯ Male
- ◯ Female
- ◯ Other

* 13. Please tell us your ethnicity:

- ◯ Asian (British, Pakistani, Indian, Bangladeshi or other)
- ◯ Black (British, African, Caribbean or other)
- ◯ White (British, Irish or other)
- ◯ Mixed ethnicity
- ◯ Chinese
- ◯ Other

* 14. Please tell us your age (GROUP SELECTION)

- ◯ Under 18
- ◯ 18-24
- ◯ 25-34
- ◯ 35-44
- ◯ 45-54
- ◯ 55-64
- ◯ 65+

* 15. Occupation

- Porter/ Cleaner/ Domestic staff
- Nurse/ Nurse trainee
- Allied health (physio/ OT/SLT/ Physician associate)
- Doctor in training
- Doctor (Senior)
- Health Care Assistant
- Administrator/ management/ secretary
- Security/ estates/ catering
- Biomedical Scientist/ other support staff
- Nurse assistance
- Volunteer
- Volunteer
- Other

* 16. Have you been working in a ‘COVID facing’ patient area?

- Yes
- No

* 17. Where was your normal place of work during the COVID pandemic?

- Not in a clinical area
- COVID designated Ward
- Non-COVID designated ward
- ICU/HDU
- AMU/ Emergency Department
- Multiple areas

* 18. Have you had significant contact with COVID 19 outside of work? (Please select as many as apply)

- [ ] No
- [ ] Yes, household contact confirmed case
- [ ] Yes, household contact possible case
- [ ] Yes, other contact with confirmed case
- [ ] Yes, other contact with possible case

* 19. Have you ever felt symptomatic with COVID 19?

- ◯ Yes
- ◯ No

* 20. What was the approximate date of your infection?

Date / Time

Date

MM/DD/YYYY

* 21. How long did your symptoms last?

- <24 hours
- 1-2 days
- 3-7 days
- 1-2 weeks
- more than 2 weeks

* 22. Did you have the classic symptoms of high fever and persistent cough for several days?

- Yes
- No

* 23. What was your temperature in degrees C?

- ◯ Less than 37.5
- ◯ 37.5 - 37.7
- ◯ 37.8 - 38.0
- ◯ 38.1 - 38.4
- ◯ 38.5 - 38.9
- ◯ 39.0 - 39.5
- ◯ More than 39.5
- ◯ I didn't check my temperature

* 24. If you had a cough did you have a persistent cough i.e. coughing for more than an hour, or 3 or more coughing episodes in 24hours?

- ◯ Yes
- ◯ No

* 25. Did you experience unusual fatigue?

- ◯ Yes
- ◯ No

* 26. Did you have a headache?

- ◯ Yes
- ◯ No

* 27. Did you experience unusual shortness of breath?

- ◯ Yes - mild
- ◯ Yes - significant
- ◯ Yes - severe
- ◯ No

* 28. Did you have a sore throat?

- ◯ Yes
- ◯ No

* 29. Did you have a loss of smell or taste?

- ◯ Yes
- ◯ No

* 30. Did you have an unusually hoarse voice?

- ◯ Yes
- ◯ No

* 31. Did you experience any unusual chest pain or tightness?

- ◯ Yes
- ◯ No

* 32. Did you have any unusual abdominal pain?

- ◯ Yes
- ◯ No

* 33. Did you have any diarrhoea?

- ◯ Yes
- ◯ No

* 34. Did you have unusual strong muscle pain?

- ◯ Yes
- ◯ No

* 35. Did you have any confusion/disorientation/drowsiness?

- ◯ Yes
- ◯ No

* 36. Did you have a reduced appitite (skip meals)?

- ◯ Yes
- ◯ No

37. Are there any other important symptoms you want to share?

* 38. Were you classified by your GP, specialist, or the hospital occupational health service as higher risk of COVID-19 because of health problems?

- ◯ Yes
- ◯ No

Your Health and Well-Being

This survey asks for your views about your health. This information will help you keep track of how you feel and how well you are able to do your activities. For each of the following questions, please mark the box which best describes your answer.

Copyright SF12v2 (r) Health Survey (c) 1994,2002 Medical Outcomes Trust and QualityMetric Incorporated. All rights reserved.

* 39. In general, would you say your health is:

Excellent	Very good	Good	Fair	Poor
○	○	○	○	○

* 40. The following questions are about activities you might do during a typical day. Does your health now limit you in these activities? If so, how much?

	Yes, limited a lot	Yes, limited a little	No, not limited at all
Moderate activities such as moving a table, pushing a vacuum cleaner, bowling, or playing golf	○	○	○
Climbing several flights of stairs	○	○	○

* 41. During the past 4 weeks how much of the time have you had any of the following problems with your work or other regular daily activities as a result of your physical health?

	All of the time	Most of the time	Some of the time	A little of the time	None of the time
a.) Accomplished less than you would like	○	○	○	○	○
b.) Were limited in the kind of work or other activities	○	○	○	○	○

* 42. During the past 4 weeks how much of the time have you had any of the following problems with your work or other regular daily activities as a result of any emotional problems (such as feeling depressed or anxious)?

	All of the time	Most of the time	Some of the time	A little of the time	None of the time
a.) Accomplished less than you would like	○	○	○	○	○
b.) Did work or other activities less carefully than usual	○	○	○	○	○

* 43. During the past 4 weeks, how much did pain interfere with your normal work (including both work outside the home and housework)?

Not at all	A little bit	Moderately	Quite a bit	Extremely
○	○	○	○	○

* 44. These questions are about how you feel and how things have been with you during the past 4 weeks. For each question, please give the one answer that comes closest to the way you have been feeling. How much of the time during the past 4 weeks...

	All of the time	Most of the time	Some of the time	A little of the time	None of the time
a) Have you felt calm and peaceful?	○	○	○	○	○
b) Did you have a lot of energy?	○	○	○	○	○
c) Have you felt downhearted and low?	○	○	○	○	○

* 45. During the past 4 weeks how much of the time has your physical health or emotional problems interfered with your social activities (like visiting with friends, relatives, etc.)?

All of the time	Most of the time	Some of the time	A little of the time	None of the time
○	○	○	○	○

* 46. How often have they been bothered by the following over the past 2 weeks?

	Not at all	Several days	More than half the days	Nearly every day
Feeling nervous, anxious or on edge	○	○	○	○
Not being able to stop or control worrying	○	○	○	○
Worrying too much about different things	○	○	○	○
Having trouble relaxing	○	○	○	○
Being so restless that it is hard to sit still	○	○	○	○
Becoming easily annoyed or irritable	○	○	○	○
Feeling afraid, as if something awful might happen	○	○	○	○

Many Thanks!
You have completed the survey. We greatly appreciate your time and support in this study.
