# Supplementary material for: COVID-19 seroprevalence after the first UK wave of the pandemic and its association with the physical and mental wellbeing of secondary care healthcare workers
Source: Brain Behav Immun Health. 2022 Aug 6;24:100492. doi: 10.1016/j.bbih.2022.100492 (PMC9355737; doi:10.1016/j.bbih.2022.100492)
Supplement: Multimedia component 3 [file mmc3.docx]

CHIP

COVID 19 Health Professional Impact Study

Version-7 dated16/06/2020

SPONSOR: Royal Wolverhampton NHS Trust

**Study Management Group**

Co-Chief Investigators: Prof S Basu, The Royal Wolverhampton NHS Trust

Co-investigators: Prof J Cotton, The Royal Wolverhampton NHS Trust

Prof M Brookes, The Royal Wolverhampton NHS Trust

Dr J Bateman, The Royal Wolverhampton NHS Trust

Dr H Morrisey, University of Wolverhampton

Dr Claire Ford, The Royal Wolverhampton NHS Trust

Statistician: Prof A Nevill, University of Wolverhampton

Study Management: Mr A Smallwood, The Royal Wolverhampton NHS Trust

**Sponsor**

The Royal Wolverhampton Trust is the research sponsor for this study. For further information regarding the sponsorship conditions, please contact R&D Department, The Royal Wolverhampton NHS Trust– Tel: 01902 695065 or email: [sarah.glover7@nhs.net](mailto:sarah.glover7@nhs.net)

**Funder**

TBA

This protocol describes the CHIP study and provides information about procedures for entering participants. Every care was taken in its drafting, but corrections or amendments may be necessary. These will be circulated to investigators in the study.

Problems relating to this study should be referred, in the first instance, to the Chief Investigator.

This study will adhere to the principles outlined in the UK Policy Framework for Health and Social Care Research ver. 3.0 (2017). It will be conducted in compliance with the protocol, the Data Protection Act and other regulatory requirements as appropriate.

**Table of Contents**

[1. INTRODUCTION 5](#_Toc42699621)

[1.1 BACKGROUND 5](#_Toc42699622)

[2. STUDY OBJECTIVES 6](#_Toc42699623)

[3. STUDY DESIGN 7](#_Toc42699624)

[3.1 Study outcome measures 7](#_Toc42699625)

[4. Participant Entry 8](#_Toc42699626)

[4.1 Inclusion Criteria 8](#_Toc42699627)

[4.2 EXCLUSION CRITERIA 8](#_Toc42699628)

[4.3 WithdrawAl criteria 8](#_Toc42699629)

[5. ASSESSMENT AND FOLLOW-UP 8](#_Toc42699630)

[6. STATISTICs and data analysis 10](#_Toc42699631)

[7. regulatory issues 10](#_Toc42699632)

[7.1 Ethics AND REGULATORY approval 10](#_Toc42699633)

[7.2 Consent 10](#_Toc42699634)

[7.3 Confidentiality 11](#_Toc42699635)

[7.4 Indemnity 11](#_Toc42699636)

[7.5 Sponsor 11](#_Toc42699637)

[7.6 Funding 11](#_Toc42699638)

[7.7 Audits and Inspections 11](#_Toc42699639)

[8. Study Management 11](#_Toc42699640)

[9. Publication Policy 12](#_Toc42699641)

[10. References 12](#_Toc42699642)

**Glossary of Abbreviations and technical terms**

|  |  |
| --- | --- |
| RT-PCR | Real-time reverse transcriptase-polymerase chain reaction |
| PPE | Personal protective equipment |
| BAME | Black and minority ethnic groups |
| NIHR | National institute of healthcare research |
| ONS | Office of National Statistics |
| UKRI | UK research and innovation |
| SARS-CoV-2 | Severe acute respiratory syndrome coronavirus 2 |
| IMD | Index of multiple deprivation |
| RWT | The Royal Wolverhampton NHS Trust |
| SMS | Short message service (text message) |
| R&D | Research & Development |
| SOP | Standard Operating Procedure |
| HR | Human Resources |
|  |  |
|  |  |
|  |  |
|  |  |
|  |  |
|  |  |
|  |  |
|  |  |
|  |  |
|  |  |
|  |  |
|  |  |
|  |  |
|  |  |
|  |  |
|  |  |
|  |  |
|  |  |

**Keywords**

Coronavirus

COVID-19

Antibody

Test

**Study Summary**

| **TITLE** | A pilot assessment of the COVID 19 antibody status and associated factors, in individuals employed by a large acute NHS trust during the Coronavirus pandemic  **SHORT STUDY TITLE / ACRONYM:**  COVID-19 health professional impact study (**CHIP**) |
| --- | --- |
| **DESIGN** | Observational retrospective analysis. |
| **AIMS** | To determine the factors associated with antibody positivity in employees of a large NHS trust having passed through the first peak of the COVID-19 pandemic |
| **OUTCOME MEASURES** | Proportion of antibody positive participants, associations with workplace, work type, demographics and behavioural factors, ethnicity, and symptoms |
| **POPULATION** | NHS Trust employees self referring for routine COVID -19 antibody testing |
| **ELIGIBILITY** | All employees tested for Coronavirus antibodies |
| **duration** | 1 year 3 months  Recruitment period of 3 months with participation duration of 1 year. |

# 1. INTRODUCTION

## BACKGROUND

The COVID-19 pandemic has had a huge impact on healthcare resources and staff in the UK. Understanding the key risk factors associated with infection amongst healthcare workers is essential for future pandemic response plans. Currently there are scarce data relating to the infection rates and associated factors amongst healthcare workers in the UK.

Whilst many of the healthcare challenges posed by the current pandemic have centred around a shortage of physical resources (access to ventilators, hospital beds, PPE availability), these are being remedied with improvements in manufacture and supply. However, Human Resources are less easy to replace and the impact of COVID -19 has been disproportionately high on healthcare workers, with a documented 106 individuals dying in the UK in April 2020 alone.

Studies of infection rates in healthcare workers have largely relied on the real-time reverse transcriptase-polymerase chain reaction (RT-PCR) test to date.

One study of 50% of staff at the privately run Portland hospital showed that at the height of the pandemic, 18% of staff tested positive, with a higher degree of positive results in patient-facing staff^1^.. Interestingly they showed that staff living in more socially deprived areas were significantly more likely to test positive for the virus.

Healthcare workers are twice as likely to succumb to Coronavirus infection, when compared to the general population and those from Black and minority ethnic (BAME) backgrounds appear to be particularly at risk (ONS statistics).

In contrast, the COVIDSORTIUM study, conducted at a large London teaching hospital reported a peak infection rate amongst staff of 7.1% falling rapidly over following weeks to approximately 1% suggesting that infection amongst health workers reflected that of the general population^2^.

UK research and innovation (UKRI) and National institute of healthcare research (NIHR) have recently put out a call for research into the risk factors, transmission and prevalence of severe acute respiratory syndrome coronavirus 2 (SARS-CoV-2).

Currently there is no evidence that the presence of SARS-CoV-2 antibodies provides long term immunity to future infection. In this study we will follow subjects at six months and one year to determine new infection rates in those with antibodies and those without at the time of initial testing.

In this project we aim to describe the prevalence of SARS-CoV-2 antibody presence in an acute NHS hospital that was particularly hard hit by the first wave of the pandemic and relate prevalence rate amongst staff to:

- Patient Facing vs Non Patient facing
- Previous RT-PCR test
- Previous RT-PCR test positive
- Symptomatic in past vs asymptomatic
- Types of symptoms reported
- Time lapse between reported symptoms and SARS-CoV-2 antibody test
- Age
- Ethnicity
- Index of multiple deprivation (IMD) from Postcode
- Previous period of self isolation or not
- Degree of anxiety regarding infection with virus
- Presence of one or more high risk factors for COVID 19

In addition we will relate the presence or absence of SARS-CoV-2 antibody at the time of testing to the future incidence of clinical COVID-19 infection^3^. The significance of antibody presence and if it confers protection to future sars-Cov2 infection is unknown.

**1.2 RATIONALE FOR CURRENT STUDY**

The current study aims to detail the current level of SARS-CoV-2 antibody positivity and thereby infer the likely risk to healthcare workers in the UK to COVID-19 infection^3^. We will determine whether certain characteristics (detailed above) will have an impact on likelihood of infection and antibody response. Furthermore we will determine the impact of the presence of antibodies on the likelihood of future clinical infection over a 12 month period.

# 2. STUDY OBJECTIVES

To determine:

1. The prevalence of SARS-CoV-2 antibody positivity in healthcare staff in an acute NHS trust that has passed through the first peak of Coronavirus infection
2. To assess the relative importance of
   1. Job role
   2. Age
   3. Ethnicity
   4. Previous RT-PCR test status
   5. Previous clinical infection
   6. Index of multiple deprivation
   7. Previous period of self isolation
   8. Level of anxiety regarding COVID 19
   9. Presence of one or more high risk factors for COVID 19
   10. Presence or absence of self-reported previous COVID 19 symptoms
3. To assess whether antibody positivity reduces risk of clinical SARS-CoV-2 infection in the following 12 months.

# 3. STUDY DESIGN

This observational study will utilise the results of the Abbott SARS-CoV-2 IgG assay analysed on either Abbott Architect i2000sr or Alinity ci (Abbott Diagnostics, Abbott Park, IL, USA) and in addition consent will be gained to retrieve and analyse data held by the Trust in relation to this test.

Subjects will be invited to take part, by SMS, via the HR department, in a smart - phone based questionnaire to gain additional data as detailed above.

All employees of the Royal Wolverhampton Trust (RWT) requesting an antibody test for SARS-CoV-2 will be approached to be part of the study – it is estimated that this will be up to 4000 individuals and we aim for a response from 3000 participants.

The study will also be advertised via trust communication methods – the screen saver, the weekly bullet-in, email to all through comms. This will act as a reminder for staff who have had the antibody testing to complete the questionnaire via the SMS link, but also offer an alternative method to access the online PIS, consent and complete the questionnaire if they did not provide a mobile telephone number for the SMS main recruitment method.

Consent for inclusion to the study will be gained by the smartphone link that will then lead on to the questionnaire (survey monkey). If they have not provided a mobile number to enable SMS, alternative method of contact such as email or letter will be used.

If after sending the SMS the questionnaire has not been completed after 2 weeks, a reminder SMS will be sent out.

A further follow up questionnaire will be sent at six and twelve months to determine clinical COVID status by SMS (or alternative method if mobile number not provided).

Data will be collected, linked, de-identified and stored on a dedicated database, on RWT trust servers. Data processing will be performed by a dedicated clinical trial assistant (CTA). Study data will only be accessible by the CTA, study manager or chief investigators. The data will be retrieved, and pseudoanonymised data will be analysed by the investigators and study statistician.

## 3.1 Study outcome measures

Primary Outcome:

- To identify the proportion of employees tested who have evidence of SARS-CoV-2 antibodies
- To identify factors associated with a positive test
  - Type of healthcare role
  - Ethnicity
  - Age
  - Index of multiple deprivation
  - Previous RT-PCR test status
  - Previous clinical COVID infection
  - Previous period of self isolation
  - Level of COVID-19 related anxiety
  - Presence of one or more high risk factors for COVID 19
  - Presence or absence of self-reported COVID 19 symptoms
- To assess the likelihood of clinically relevant (causing illness) infection with SARS-CoV-2in subjects with both positive and negative SARS-CoV-2 antibody tests.

# 4. Participant Entry

This study will include members of staff self referring to have the SARS-CoV-2 antibody test at RWT.

## 4.1 Inclusion Criteria

- All Staff employed by RWT, having undergone the SARS-CoV-2antibody test at the Trust’s testing facility will be eligible for inclusion in the study irrespective of antibody status.

## 4.2 EXCLUSION CRITERIA

- Royal Wolverhampton NHS Trust staff who have not participated in antibody testing
- Staff who have opted out to receiving the SMS invite and be part of the study

## 4.3 WithdrawAl criteria

Subjects wishing to withdraw from the study, once enrolled can do so at any time without giving a reason. All of their data stored in association with the current study will be erased and not used in the final analysis.

# 5. ASSESSMENT AND FOLLOW-UP

Potential participants will be identified by the Occupational Therapy department once they have undergone the SARS-CoV-2 antibody test at RWT as part of routine Trust practice.

As part of the clinical service, participants are asked to provide a number of details and sign a form to confirm that they understand the implications of having the test. They will provide:

ESR/employment number

Name

DoB

First line of address

Postcode

Mobile phone number

This data will be stored with Occupational Health and the HR department.

The Occupational Health team will notify the HR team of staff receiving antibody testing and then the HR team will contact potential participants by SMS (or alternative method if mobile number not given) to invite them to be part of the study. The potential participant will consent to be sent the link via return SMS before sending the link to the questionnaire by the same method. Staff will have the opportunity to opt-out of receiving the SMS invite if they so wish, by notifying the HR team in response to the advertisements.

The participant will read the online PIS and give electronic consent to participate and then complete the online questionnaire. Once completed, the questionnaire answers will be held on a database and be linked through the mobile number and employment number to obtain the antibody test result and the other data as listed above provided at testing.

A 6 and 12 month follow up questionnaire will be requested to be completed by SMS link to the participants by the researchers. Again, a reminder will be sent after 2 weeks if the participant has not completed the follow-up questionnaire.

There will be no payments to participants as the questionnaires are online.

**Data Collection – Questionnaire:**

Data to be collected from the questionnaire includes:

- Demographics such as Ethnicity, Age Group, Occupation, notification of patient-facing role
- Symptomatic details
- High-risk classification
- Wellbeing status through elements of the validated SF12 and the GAD7 questionnaires

The same questionnaire will be used at all time-points.

**Study Schedule:**

| **Procedures** |  |  |  |  |
| --- | --- | --- | --- | --- |
|  | **Screening** | **Baseline** | **6 Months** | **12 Months** |
| Consent for SMS link | X |  |  |  |
| Informed consent (online) |  | X |  |  |
| Initial questionnaire |  | X |  |  |
| Linkage to HR data |  | X |  |  |
| Linkage to antibody test |  | X |  |  |
| Questionnaire - follow up |  |  | X | X |

The study will end when all RWT employees have had the antibody test and been contacted to be part of the study or 3,000 subjects are recruited, whichever is sooner.

# 6. STATISTICs and data analysis

We aim to analyse data on all subjects taking the test. It is not an interventional trial and therefore a power calculation is not required. Previous studies have been in the low hundreds – we aim to rapidly provide data on the largest acute trust population yet studied.

Current early test results indicate a positive rate of 16% - 3,000 tests will therefore yield 480 positive subjects.

Statistical analysis will be largely descriptive. Factors associated with test positivity will be determined using cox proportional-hazard regression analysis.

Data and all appropriate documentation will be stored for a minimum of 5 years after the completion of the study, including the follow-up period. Storage will be off-site through the archiving facility used by the Sponsor.

# 7. regulatory issues

#

## 7.1 Ethics AND REGULATORY approval

The Chief Investigator will/ has obtained approval from an approved Research Ethics Committee and the HRA.

This study is seen as low risk.

The sample for antibody testing has been taken as standard practice and results are available. The researchers will need to link the antibody test result and data held by HR to the participant questionnaire data. This will only be done after informed consent is given.

There is potential that the questionnaire could identify issues and/or extra support that may be needed for the participant. The researchers have a duty of care to signpost for support or counselling as required and participants will be made aware of this in the participant information sheet where participants will be provided with links to the Trust Mental Wellness online support pages and support links.

## 7.2 Consent

No data will be collected from participants that do not consent to the study.

Adverts will be distributed through methods outlined above where staff will be given the links to complete the online consent form for the matching of the questionnaire answers with the antibody test results and HR data.

Mobile numbers to send SMS are held with the HR department and HR will send out the initial request and link to first questionnaire on behalf of the researchers, therefore the researchers will not have access to personal data until consent has been given.

Consent for the study will be online as this study is a data linkage and questionnaire study which involves no extra visits to site for the study or extra invasive procedures.

## 7.3 Confidentiality

Participant confidentiality will be maintained and the study will be compliant with the requirements of the Data Protection Act 2018. All study data will be pseudonymised with a study code and identifiable data such as mobile number and Employment number will be stored separately to study data to allow linkage of the questionnaire. Only post-code will form part of the dataset. The data will be stored in encrypted digital files within password protected folders and storage media. Access will be limited to the minimum number of individuals necessary for quality control, audit, and analysis. No personal data other than post-code will be transferred off-site for analysis. The Chief Investigator will act as custodian of the data.

All investigators and study site staff must comply with the requirements of the Data Protection Act 2018 with regards to the collection, storage, processing and disclosure of personal information and will uphold the Act’s core principles.

## 7.4 Indemnity

Standard NHS Indemnity will apply for participants recruited into this study to meet the potential legal liability of the sponsor for harm to participants arising from the management of the research, the design of the research and the conduct of the research.

## 7.5 Sponsor

The Royal Wolverhampton NHS Trust will act as Sponsor.

## 7.6 Funding

TBC

## 7.7 Audits and Inspections

The Royal Wolverhampton NHS Trust through the R&D Directorate will make a decision on any monitoring plan for this study and level of requirement based on their SOPs for monitoring.

# 8. Study Management

The day-to-day management of the study will be co-ordinated by Andrew Smallwood with oversite from the Chief Investigator and Collaborator.

A Study Steering Group will review the study on a regular ongoing basis to ensure the study is running to plan.

A number of staff have reviewed the study design and questionnaire for acceptability and ease of use.

# 9. Publication Policy

The Royal Wolverhampton NHS Trust as Sponsor owns the data arising from the study.

On completion of the study, the data will be analysed and tabulated and a Final Study Report prepared. The results will be published in a reputable journal.

Participants will be notified of the outcome of the study by provision of the publication, and through the Trust channels of communication.

# 10. References

1. Khalistan et al Lancet, May 18^th^,doi: 51473-3099(20)30403-5
2. Trimble T et al Lancet, May 2020 50140-6736(20)31100-4
3. <https://www.the-scientist.com/news-opinion/what-do-antibody-tests-for-sars-cov-2-tell-us-about-immunity--67425>
4. Mao L, Jin H, Wang M, et al. Neurologic Manifestations of Hospitalized Patients With Coronavirus Disease 2019 in Wuhan, China. JAMA Neurol. 2020;77(6):683–690. doi:10.1001/jamaneurol.2020.1127
5. Mizumoto K, Kagaya K, Zarebski A, *et al.* Estimating the asymptomatic proportion of coronavirus disease 2019 (COVID-19) cases on board the Diamond Princess cruise ship, Yokohama, Japan, 2020. *Euro Surveill* 2020;**25**.
   doi:[10.2807/1560-7917.ES.2020.25.10.2000180](http://dx.doi.org/10.2807/1560-7917.ES.2020.25.10.2000180)
6. [Day M. Covid-19: identifying and isolating asymptomatic people helped eliminate virus in Italian village. *BMJ* 2020;**368**:m1165.](https://www.bmj.com/content/368/bmj.m1165)
7. [Al-Tawfiq JA. Asymptomatic coronavirus infection: MERS-CoV and SARS-CoV-2 (COVID-19). *Travel Med Infect Dis* 2020;:101608.](https://www.sciencedirect.com/science/article/pii/S1477893920300752?via%3Dihub)
8. Zhou X, Li Y, Li T, *et al.* Follow-up of the asymptomatic patients with SARS-CoV-2 infection. *Clin Microbiol Infect* Published Online First: 28 March 2020.
   doi:[10.1016/j.cmi.2020.03.024](http://dx.doi.org/10.1016/j.cmi.2020.03.024)
9. [Nishiura H, Kobayashi T, Suzuki A, *et al.* Estimation of the asymptomatic ratio of novel coronavirus infections (COVID-19). *Int J Infect Dis* Published Online First: 13 March 2020.](https://www.ijidonline.com/article/S1201-9712(20)30139-9/pdf) d[oi:](http://paperpile.com/b/iO99Sg/eS3t)[10.1016/j.ijid.2020.03.020](http://dx.doi.org/10.1016/j.ijid.2020.03.020)
10. [Kimball A, Hatfield KM, Arons M, *et al.* Asymptomatic and Presymptomatic SARS-CoV-2 Infections in Residents of a Long-Term Care Skilled Nursing Facility – King County, Washington, March 2020. *MMWR Morb Mortal Wkly Rep* 2020;**69**:377–81.](https://www.ncbi.nlm.nih.gov/research/coronavirus/publication/32240128)
11. Quilty BJ, Clifford S, Flasche S, *et al.* Effectiveness of airport screening at detecting travellers infected with novel coronavirus (2019-nCoV). Eurosurveillance. 2020;**25**.
    doi:[10.2807/1560-7917.es.2020.25.5.2000080](http://dx.doi.org/10.2807/1560-7917.es.2020.25.5.2000080)
12. [Tian S, Hu N, Lou J, *et al.* Characteristics of COVID-19 infection in Beijing. *J Infect* 2020;**80**:401–6.](https://www.ncbi.nlm.nih.gov/research/coronavirus/publication/32112886)
13. [Sun WW, Ling F, Pan JR, *et al.* [Epidemiological characteristics of 2019 novel coronavirus family clustering in Zhejiang Province]. *Zhonghua Yu Fang Yi Xue Za Zhi* 2020;**54**:E027.](https://www.ncbi.nlm.nih.gov/research/coronavirus/publication/32171192)
14. Han Y, Yang H. The transmission and diagnosis of 2019 novel coronavirus infection disease (COVID-19): A Chinese perspective. *J Med Virol* Published Online First: 6 March 2020. doi:[10.1002/jmv.25749](http://dx.doi.org/10.1002/jmv.25749)
15. Qiu H, Wu J, Hong L, *et al.* Clinical and epidemiological features of 36 children with coronavirus disease 2019 (COVID-19) in Zhejiang, China: an observational cohort study. *Lancet Infect Dis* Published Online First: 25 March 2020.
    doi:[10.1016/S1473-3099(20)30198-5](http://dx.doi.org/10.1016/S1473-3099(20)30198-5)
16. [Anastassopoulou C, Russo L, Tsakris A,](https://journals.plos.org/plosone/article?id=10.1371/journal.pone.0230405) *[et al.](https://journals.plos.org/plosone/article?id=10.1371/journal.pone.0230405)* [Data-based analysis, modelling and forecasting of the COVID-19 outbreak.](https://journals.plos.org/plosone/article?id=10.1371/journal.pone.0230405) *[PLoS One](https://journals.plos.org/plosone/article?id=10.1371/journal.pone.0230405)* [2020;](https://journals.plos.org/plosone/article?id=10.1371/journal.pone.0230405)**[15](https://journals.plos.org/plosone/article?id=10.1371/journal.pone.0230405)**[:e0230405.](https://journals.plos.org/plosone/article?id=10.1371/journal.pone.0230405)
17. [Day M. Covid-19: four fifths of cases are asymptomatic, China figures indicate. *BMJ* 2020;**369**:m1375.](https://www.bmj.com/content/369/bmj.m1375)
18. Hu Z, Song C, Xu C, *et al.* Clinical characteristics of 24 asymptomatic infections with COVID-19 screened among close contacts in Nanjing, China. Science China Life Sciences. 2020. doi:[10.1007/s11427-020-1661-4](http://dx.doi.org/10.1007/s11427-020-1661-4)
19. Newman T. COVID-19: Study estimates rate of ‘silent transmission’. Medical News Today. 2020.<https://www.medicalnewstoday.com/articles/covid-19-study-estimates-rate-of-silent-transmission> (accessed 5 Apr 2020).
20. Song H, Xiao J, Qiu J, *et al.* A considerable proportion of individuals with asymptomatic SARS-CoV-2 infection in Tibetan population.
    doi:[10.1101/2020.03.27.20043836](http://dx.doi.org/10.1101/2020.03.27.20043836)
21. Q&A: Similarities and differences – COVID-19 and influenza. <https://www.who.int/news-room/q-a-detail/q-a-similarities-and-differences-covid-19-and-influenza> (accessed 5 Apr 2020).
22. Renwick D. Have I already had coronavirus? How would I know and what should I do? the Guardian. 2020.<http://www.theguardian.com/us-news/2020/apr/05/have-i-already-had-coronavirus-how-would-i-know> (accessed 5 Apr 2020).
23. Otmani M. COVID-19: First results of the voluntary screening in Iceland – Nordic Life Science – the leading Nordic life science news service. Nordic Life Science – the leading Nordic life science news service. 2020.<https://nordiclifescience.org/covid-19-first-results-of-the-voluntary-screening-in-iceland/> (accessed 5 Apr 2020)
24. [NPR Choice page.](http://paperpile.com/b/iO99Sg/R19c) <https://www.npr.org/sections/health-shots/2020/03/31/824155179/cdc-director-on-models-for-the-months-to-come-this-virus-is-going-to-be-with-us?t=1586065796492> [(accessed 5 Apr 2020).](http://paperpile.com/b/iO99Sg/R19c)
25. CDC. Coronavirus Disease 2019 (COVID-19). Centers for Disease Control and Prevention. 2020.[https://www.cdc.gov/coronavirus/2019-ncov/hcp/clinical-guidance-management-patients.html#Asymptomatic](https://www.cdc.gov/coronavirus/2019-ncov/hcp/clinical-guidance-management-patients.html#Asymptomatic) (accessed 5 Apr 2020).
26. Coronavirus, Castiglione d’Adda è un caso di studio: ‘Il 70% dei donatori di sangue è positivo’. lastampa.it. 2020.<https://www.lastampa.it/topnews/primo-piano/2020/04/02/news/coronavirus-castiglione-d-adda-e-un-caso-di-studio-il-70-dei-donatori-di-sangue-e-positivo-1.38666481> (accessed 5 Apr 2020).
