# Supplementary material for: COVID-19 seroprevalence after the first UK wave of the pandemic and its association with the physical and mental wellbeing of secondary care healthcare workers
Source: Brain Behav Immun Health. 2022 Aug 6;24:100492. doi: 10.1016/j.bbih.2022.100492 (PMC9355737; doi:10.1016/j.bbih.2022.100492)
Supplement: Multimedia component 2 [file mmc2.pdf]

East of England - Cambridge South Research Ethics Committee
The Old Chapel
Royal Standard Place
Nottingham
NG1 6FS

<u>Please note</u>: This is the favourable opinion of the REC only and does not allow you to start your study at NHS sites in England until you receive HRA Approval

18 August 2020

Professor S Basu
Professor of Haematology
The Royal Wolverhampton NHS Trust
Haematology Department, New Cross Hospital
Wolverhampton
WV10 0QP

Dear Professor Basu,

**Study title:**	**A pilot assessment of the COVID 19 antibody status and associated factors, in individuals employed by a large acute NHS trust during the Coronavirus pandemic**
**REC reference:**	**20/EE/0201**
**Protocol number:**	**2020COV112**
**IRAS project ID:**	**287432**

Thank you for your letter of 17 August 2020, responding to the Proportionate Review Sub-Committee's request for changes to the documentation for the above study.

The revised documentation has been reviewed and approved on behalf of the Chair.

Confirmation of ethical opinion

On behalf of the Committee, I am pleased to confirm a favourable ethical opinion for the above research on the basis described in the application form, protocol and supporting documentation as revised.

Conditions of the favourable opinion

The REC favourable opinion is subject to the following conditions being met prior to the start of the study.

Confirmation of Capacity and Capability (in England, Northern Ireland and Wales) or NHS management permission (in Scotland) should be sought from all NHS organisations involved in the study in accordance with NHS research governance arrangements. Each NHS organisation must confirm through the signing of agreements and/or other documents that it has given permission for the research to proceed (except where explicitly specified otherwise).

Guidance on applying for HRA and HCRW Approval (England and Wales)/ NHS permission for research is available in the Integrated Research Application System.

For non-NHS sites, site management permission should be obtained in accordance with the procedures of the relevant host organisation.

Sponsors are not required to notify the Committee of management permissions from host organisations.

Registration of Clinical Trials

It is a condition of the REC favourable opinion that **all clinical trials are registered** on a publicly accessible database. For this purpose, 'clinical trials' are defined as the first four project categories in IRAS project filter question 2. Registration is a legal requirement for clinical trials of investigational medicinal products (CTIMPs), except for phase I trials in healthy volunteers (these must still register as a condition of the REC favourable opinion).

Registration should take place as early as possible and within six weeks of recruiting the first research participant at the latest. Failure to register is a breach of these approval conditions, unless a deferral has been agreed by or on behalf of the Research Ethics Committee (see here for more information on requesting a deferral: https://www.hra.nhs.uk/planning-and-improving-research/research-planning/research-registration-research-project-identifiers/

As set out in the UK Policy Framework, research sponsors are responsible for making information about research publicly available before it starts e.g. by registering the research project on a publicly accessible register. Further guidance on registration is available at: https://www.hra.nhs.uk/planning-and-improving-research/research-planning/transparency-responsibilities/

You should notify the REC of the registration details. We routinely audit applications for compliance with these conditions.

Publication of Your Research Summary

We will publish your research summary for the above study on the research summaries section of our website, together with your contact details, no earlier than three months from the date of this favourable opinion letter.

Should you wish to provide a substitute contact point, make a request to defer, or require further information, please visit: https://www.hra.nhs.uk/planning-and-improving-research/application-summaries/research-summaries/

N.B. If your study is related to COVID-19 we will aim to publish your research summary within 3 days rather than three months.

During this public health emergency, it is vital that everyone can promptly identify all relevant research related to COVID-19 that is taking place globally. If you haven't already done so, please register your study on a public registry as soon as possible and provide the HRA with the registration detail, which will be posted alongside other information relating to your project. We are also asking sponsors not to request deferral of publication of research summary for any projects relating to COVID-19. In addition, to facilitate finding and extracting studies related to COVID-19 from public databases, please enter the WHO official acronym for the coronavirus disease (COVID-19) in the full title of your study. Approved COVID-19 studies can be found at: https://www.hra.nhs.uk/covid-19-research/approved-covid-19-research/

It is the responsibility of the sponsor to ensure that all the conditions are complied with before the start of the study or its initiation at a particular site (as applicable).

After ethical review: Reporting requirements

The attached document "After ethical review – guidance for researchers" gives detailed guidance on reporting requirements for studies with a favourable opinion, including:

- Notifying substantial amendments
- Adding new sites and investigators
- Notification of serious breaches of the protocol
- Progress and safety reports
- Notifying the end of the study, including early termination of the study
- Final report

The latest guidance on these topics can be found at https://www.hra.nhs.uk/approvals-amendments/managing-your-approval/.

Ethical review of research sites

The favourable opinion applies to all NHS/HSC sites taking part in the study, subject to management permission being obtained from the NHS/HSC R&D office prior to the start of the study (see "Conditions of the favourable opinion" above).

Approved documents

The documents reviewed and approved by the Committee are:

Document	*Version*	*Date*
Copies of advertisement materials for research participants [CHIP Advert Clean]	4	10 August 2020
Copies of advertisement materials for research participants [CHIP Advert tracked changes]	4	10 August 2020

Copies of advertisement materials for research participants [CHIP Poster tracked changes]	3	10 August 2020
Copies of advertisement materials for research participants [CHIP Poster Clean]	3	10 August 2020
IRAS Application Form [IRAS_Form_29072020]		29 July 2020
Letter from funder [Funding Approved Email]	1	27 July 2020
Letters of invitation to participant [CHIP SMS Invite]	2	07 July 2020
Letters of invitation to participant [CHIP SMS Follow-up]	1	29 July 2020
Non-validated questionnaire [CHIP study questions clean]	2	10 August 2020
Non-validated questionnaire [CHIP study questions tracked changes]	2	10 August 2020
Other [Mobile Preview]	1	02 July 2020
Other [Response to Queries]	1	10 August 2020
Other [CHIP Protocol clean]	11	10 August 2020
Other [CHIP protocol tracked changes]	11	10 August 2020
Other [Online Web Information Clean]	3	10 August 2020
Other [Online Web Information tracked changes]	3	10 August 2020
Participant consent form [CHIP Consent Form clean]	4	10 August 2020
Participant consent form [CHIP Consent form tracked changes]	4	10 August 2020
Participant information sheet (PIS) [PIS clean]	4	10 August 2020
Participant information sheet (PIS) [PIS Tracked changes]	4	10 August 2020
Referee's report or other scientific critique report [Review Approved]	1	23 July 2020
Summary CV for Chief Investigator (CI) [Basu CV]	1	17 April 2019

Statement of compliance

The Committee is constituted in accordance with the Governance Arrangements for Research Ethics Committees and complies fully with the Standard Operating Procedures for Research Ethics Committees in the UK.

User Feedback

The Health Research Authority is continually striving to provide a high quality service to all applicants and sponsors. You are invited to give your view of the service you have received and the application procedure. If you wish to make your views known please use the feedback form available on the HRA website: http://www.hra.nhs.uk/about-the-hra/governance/quality-assurance/

HRA Learning

We are pleased to welcome researchers and research staff to our HRA Learning Events and online learning opportunities– see details at: https://www.hra.nhs.uk/planning-and-improving-research/learning/

**IRAS project ID: 287432**	**Please quote this number on all correspondence**

With the Committee's best wishes for the success of this project.

Yours sincerely,

pp

Dr Leslie Gelling
Chair

Email: cambridgesouth.rec@hra.nhs.uk

Enclosures: “After ethical review – guidance for researchers”

Copy to: Mrs Sarah Glover
